# Evaluation of Three Treatments for the Resource Utilization of Cephalosporin C Fermentation Residue

**DOI:** 10.3390/toxics14030260

**Published:** 2026-03-16

**Authors:** Shengtao Ren, Wei Pu, Ruiting Fan, Yongqiang Shi, Ganggang Yang, Tianbao Ren

**Affiliations:** 1 College of Life Sciences, Henan Normal University, Xinxiang 453007, China; 2 Henan Province Engineering Laboratory for Bioconversion Technology of Functional Microbes, Xinxiang 453007, China; 3 State Key Laboratory of Cotton Biology, Institute of Cotton Research of CAAS, Anyang 455000, China; 4 Henan Biochar Engineering Technology Research Center, Tobacco College, Henan Agricultural University, Zhengzhou 450046, China

**Keywords:** cephalosporin C, cephalosporin C fermentation residue, steam explosion, composting, thermal treatment, ARGs

## Abstract

In China, antibiotic fermentation residue has been listed as a “hazardous waste” due to its high residual concentrations of antibiotics. There are many ways to deal with antibiotic fermentation residue; however, effective methods are still lacking. In the present work, steam explosion (SE), thermal, and aerobic composting treatments were performed to investigate the resource utilization of cephalosporin C fermentation residue (CFR). The results show that 0 mg/kg, 50.2 mg/kg and 150.5 mg/kg cephalosporin C (CEPC) remained after the SE, composting, and thermal treatments. The total abundance of antibiotic resistance genes (ARGs) decreased by 62.2% and 47.2% after the SE and thermal treatments and increased by 1.4 times in the samples subjected to composting. Nitrogen analysis showed that the nitrogen loss (N loss) was only 1.9% in the SE-treated samples. The antibiotic inhibition zone was reduced by 80.3%, 71.2% and 40.8% in the samples subjected to SE, composting, and thermal treatments. LC/MS showed that the β-lactam ring and dihydrothiazine ring of CEPC were largely destroyed via SE. These results suggest that the SE treatment not only decreased the residual cephalosporin and ARG levels and antimicrobial activity but also preserved most of the nitrogen. SE is therefore a feasible treatment that can be used to deal with CFR.

## 1. Introduction

Penicillin was discovered by Alexander Fleming in 1928 [1]. Since then, hundreds of antibiotics have been found, making a significant contribution to human health. Nowadays, antibiotics are widely used to cure human and animal diseases. However, the excessive use of antibiotics has led to antibiotic pollution. Approximately one-third of antibiotics are directly excreted in urine and feces from humans’ and animals’ intestinal tracts [2]. The high antibiotic concentrations remaining in these wastes are important selection pressures for surrounding microorganisms. Some bacteria with ARGs can survive in the presence of high concentrations of antibiotics, thus increasing the abundance of ARGs.

These ARGs can spread among microorganisms via gene exchange. Specifically, some horizontal transfer paths, such as conjugation, transformation, and transduction, can result in deeper gene exchange between different bacteria. Once these ARGs are transferred to human pathogenic bacteria, there will be no antibiotics available to kill them. ARG dissemination and antibiotic resistance have therefore become major threats to public and environmental health [3,4].

Antibiotic fermentation residue (AFR) is solid organic waste which is produced in the process of antibiotic production [5]. According to statistics, about 300 million tons of AFR is generated in China each year [6]. Compared with common manure, AFR contains higher residual levels of antibiotics, which could cause greater antibiotic pollution if left untreated. Therefore, AFR was listed as a hazardous solid waste in 2008, and incineration and landfilling were applied as mandatory methods to eliminate the effect of residue antibiotics on the environment [7]. However, based on the characteristics of AFR, including a high organic matter and moisture content, incineration and landfilling are rarely performed by pharmaceutical companies [8]. Establishing how to effectively deal with these solid wastes has therefore become a current research hotspot. A safe method which can both eliminate residual antibiotics and alleviate economic pressures on enterprises is desired.

An increasing number of methods have been employed to remove antibiotics and ARGs from AFR [9,10,11,12,13]. Due to their low processing costs, some biological methods have been investigated for their potential in dealing with AFR [14,15,16]. For example, in the process of biological hydrogen production, H_2_ production was greatly inhibited with the addition of CFR, with relatives of the H_2_-producing gene being significantly downregulated [17]. The yields of methane and biogas were strongly suppressed by ammonia produced by CFR [18]. Although a composting treatment was found to remove nearly 100% of penicillin, the abundance of the β-lactam resistance gene *bla_TEM_* increased twofold in the final compost [19]. Similarly, the level of *intI1*, an important integron gene, increased fivefold in samples subjected to lincomycin fermentation dregs composting compared with common sewage sludge composting [20]. However, biological treatments may be ineffective at eliminating ARGs due to the inherent antibiotic stress present in AFR.

Some physical treatments have been applied to AFR. For example, Chen [21] reported that 85.5% of cephalosporin was degraded in CFR via gamma-ray irradiation. Meanwhile, the abundance of the cephalosporin resistance gene *tolC* decreased by 74.2%. Hydrothermal pretreatment is a cleaner form of physicochemical pretreatment [22] and can effectively reduce the levels of both ARGs and mobile genetic elements in AFR. Moreover, heavy metals are also transformed into a relatively stable form [23]. However, these physical methods require either high equipment costs or huge energy costs. Furthermore, few studies have investigated the degradation products of antibiotics resulting from these physical treatments, and studies have demonstrated that antibacterial activity still persists even at low antibiotic concentrations [24]. Furthermore, some antibiotic intermediates have been found to be more toxic to microbes than their parent antibiotics [25]. After certain extreme physical treatments such as high temperatures, pressures, and radiation, the antibiotic content can be greatly reduced. However, the microbial toxicity of antibiotic intermediates may still persist. These intermediate products can still generate selection pressure on environmental microorganisms, promoting the growth of antibiotic-resistant bacteria.

SE has been used in biomass pretreatment to accelerate the degradation of lignocellulose [26,27,28]. SE involves exposing the target to high-temperature pressurized steam for a short period and then releasing the pressure suddenly. The instantaneous pressure relief process leads to an excessive pressure difference between the inside and outside of the cell, resulting in cell disruption [29]. Due to its unique mechanism of instantaneous release of high temperatures and high pressures, SE has played a crucial role in improving quality and efficiency as well as resource recycling in various industries. In the papermaking industry, for example, it can destroy the dense lignin structure of raw materials such as wood and bamboo to achieve efficient fiber dissociation [30]. Compared with traditional chemical pulping, it is green and clean. In the field of agricultural and animal husbandry, SE can break down the lignification barrier of roughage such as wheat and cotton stalks, releasing more sugars and proteins in feed [31]. Meanwhile, in the processing of Chinese medicinal materials, SE treatment can break the cell walls via precision low temperature explosion, producing more active components such as flavonoids, saponins and seaweed polysaccharides [32]. SE can convert organic solid waste from industry and agriculture into resources such as biogas and organic fertilizer [33]. For AFR, the high-temperature saturated vapors involved in SE could penetrate cells, destroy antibiotic structures, and disrupt ARGs. Meanwhile, due to the shorter reaction time, nitrogen may be maintained more effectively. However, there have been no reports of SE being used to treat AFR. It is unknown whether steam explosion can eliminate antibiotic residues and ARGs. This paper aims to compare the performance of steam explosion with that of common composing and thermal treatments in treating CFR. The reduction of antibiotics and ARGs, the loss of nutrients, antimicrobial activity, and antibiotic byproducts, and the morphology of CFR were used to evaluate the feasibility of CFR treatments. This information would be valuable for the safe disposal and treatment of AFR.

## 2. Material and Methods

### 2.1. Experimental Materials

The CFR was obtained from Jiaozuo Health Yuan Biological Products Co., Ltd. a biotechnology company in western China. The characteristics of the CFR are shown in Table 1. CEPC sodium salt (99%) was obtained from a biotechnology company in western China. The composting material, furfural slag, was obtained from a furfural enterprise. *Staphylococcus aureus* (*S. aureus*) was provided by Henan Province Engineering Laboratory for Bioconversion Technology of Functional Microbes. The ARGs and MGE primers were synthesized by Sunya Biotechnology company, Huzhou, China.

### 2.2. Experimental Setups

The steam explosion treatment of CFR was carried out using steam explosion equipment produced in the Hebi Zhengdao Machine Factory, Hebi, China. About 1.5 kg of CFR was placed into the steam chamber. The steam was pressurized up to 1.5 MP, which was maintained for 10 min, and then was suddenly released at the end of the treatment to produce an explosion effect. Thermal treatment was performed in stainless steel autoclaves at 90 °C (±5 °C) under water bath conditions for 4 h. The composting experiment was conducted in an organic fertilizer plant in western China. Furfural slag was added to adjust the C/N. The ratio of CFR to furfural slag was 3:1 to adjust the C/N to 25. The pile was turned and mixed every three days manually to provide sufficient oxygen to the microbes. The entire composting period lasted 50 days.

### 2.3. Analytic Methods

#### 2.3.1. The Detection of CEP-C

The morphology of CFR before and after each treatment was observed using a scanning electron microscope (SEM) from Hitachi Ltd. (Tokyo, Japan). The CEPC was measured using an Agilent 1200 high-performance liquid chromatograph (Agilent Technologies, Santa Clara, CA, USA) coupled with a C18 Column (5 µm, 4.6 mm × 250 mm). The mobile phase comprised methanol and 0.1% formic acid (90:10). The detection wavelength was 254 nm, and the flow rate was 1.0 ml/min with a column temperature 30 °C.

#### 2.3.2. Identification of CEP-C Degradation Products

The degradation byproducts were detected by means of HPLC coupled with an ion trap mass spectrometer. A positive-mode electrospray ionization source was chosen for MS measurements with a capillary voltage of 4.0 kV and a nebulizer temperature of 350 °C. Mass spectra were collected in the range of 110–1000 *m*/*z* using full scan mode. Firstly, CEP-C biodegradation products were identified in the total ion chromatograph by extracting *m*/*z*. Then, based on this, the product ions were further analyzed using secondary mass spectrometry. Through the characteristic peaks of secondary mass spectrometry, the degradation products of CEP-C were analyzed. Instrument control and data acquisition and evaluation were conducted with an Alliance HPLC and Waters Masslynx TM 4.0 (for MS) software. The molecular structures were assessed using Chem Draw Ultra 7.0 software. The data for the byproducts are shown in Table 2.

#### 2.3.3. DNA Extraction and qPCR

An FW-Stool/soil DNA kit was used to extract genomic DNA. The quality of DNA was measured using a NanoDrop spectrophotometer (Thermo Fisher Scientific, Shanghai, China). Real-time quantitative PCR (qPCR) was used to evaluate the abundance of ARGs. Eleven ARGs and two mobile genes (MGEs) were used in this experiment. The primer sequences and related resistance mechanisms are shown in Table 3. The qPCR reaction system comprised 2 µL of DNA template, 10 µL of 2X SybrGreen qPCR Master mix (TOLOBIO), 0.4 µL of each 10µM primer (Qingke, China), and 7.2 µL of double distilled water, with a total volume of 20 µL. The thermal cycling steps for qPCR amplification were as follows: (1) 95 °C for 30 s; (2) 95 °C for 10 s; (3) annealing temperature for 30 s; (4) 60 °C for 30 s; and (5) a plate read, where steps (2) to (4) were repeated 40 times. The absolute abundance (AA) of the ARGs was expressed as copies/g of dry compost. The relative abundance (RA) of ARGs was expressed as the ratio of AAs to the copies of 16S rRNA. The qPCR amplification was performed according to Ren [20].

#### 2.3.4. Detection of Nitrogen and Antimicrobial Activity

The concentrations of NH_4_^+^-N and NO_3_^−^-N were determined using an automatic chemical analyzer according to Jiang [38]. The total nitrogen (TN) was determined using a Kjeldahl apparatus. The organic N was calculated as TN minus NH_4_^+^-N and NO_3_^−^-N. The N loss was calculated as follows:

N loss (%) = (1 − (TN_treated_/TN_CFR_)) × 100%.

where TN_treated_ is the total nitrogen after SE, thermal, or composting treatments, while TN_CFR_ is the total nitrogen of CFR.

The antimicrobial activity of CEPC following SE, composting, and thermal treatments was evaluated by means of the standard Kirby–Bauer method using *S. aureus* as an indicator [39]. The LB agar plates were prepared and inoculated with bacterial suspensions of 10^6^–10^8^ CFU/mL. Four sterile round filter papers (diameter = 50 mm) were soaked in the extraction solution and then evenly placed on the plate. Sterile saline was used as a control. After incubation of the plates at 37 °C for 24 h, the diameter of the inhibition circle formed around the paper was determined.

### 2.4. Data Distribution and Statistical Analysis

All data were examined and evaluated using one-way analysis of variance with SPSS software (25.0). The graphs were produced by Origin 8.5, and the molecular forms were assessed with Chem Draw Ultra 7.0 software. The potential degradation pathway was analyzed and depicted using PowerPoint.

## 3. Results

### 3.1. Morphological Changes of CFR Under Different Treatments

**Figure 1:**
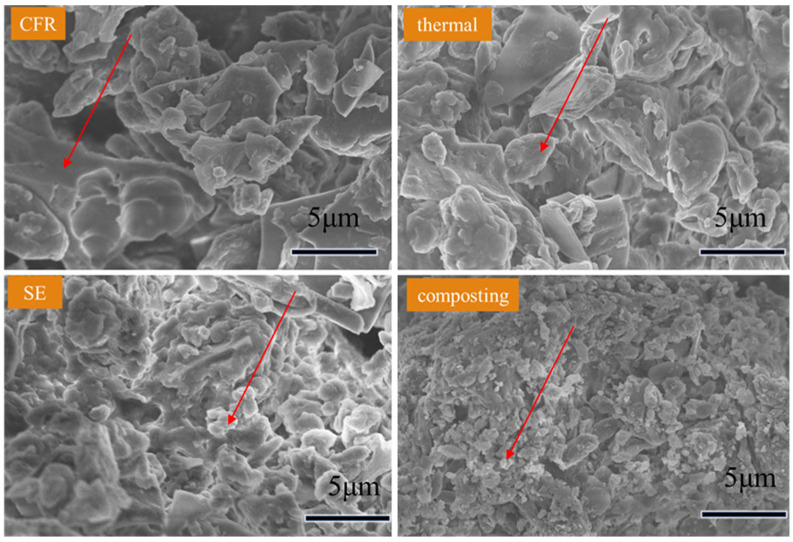
SEM pictures of the CFR and samples subject to thermal, SE, and composting treatment.

The production strain of cephalosporin is *Cephalosporium acremonium*, which is a fungus with a filamentous structure [40]. As shown in Figure 1, after the plate and frame filtering process, these filamentous structures in CFR were destroyed and compressed into smooth and regular sheet structures (red arrows). The morphology of CFR changed greatly after SE treatment. Lots of holes, debris and bumps (red arrows) were observed on the surface of the CFR, while only enlarged cells and rough surfaces (red arrows) were found after the thermal treatment. The SE process can be divided into two phases: the pressure maintenance phase and the explosion phase. The pressure maintenance phase is equivalent to thermal treatment, and the explosion phase converts the thermal energy into mechanical energy, which causes cells to break down into small pieces [41]. So, compared with thermal treatment, SE causes greater damage to CFR. After SE treatment, much intracellular organic matter was released, which was more conducive to soil microbial utilization [42]. In the composted samples, many cocci and rod microorganisms (red arrows) could be found on the surface of the CFR. These microbes might feed on CFR, despite the presence of high contents of CEPC in CFR [43].

### 3.2. The Reduction of Antibiotic and ARGs

**Figure 2:**
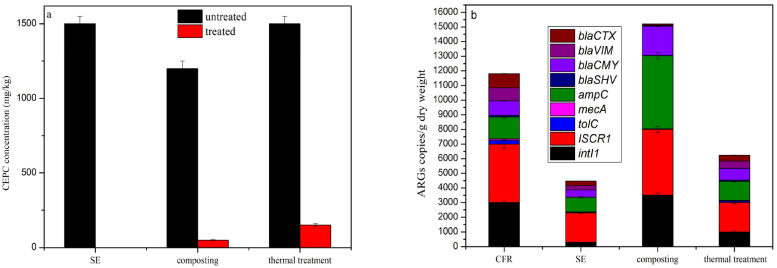
Removal of CEPC (**a**) and ARGs (**b**) after SE, composting, and thermal treatments.

The changes in CEPC following the composting, thermal, and steam explosion treatments are shown in Figure 2a. The initial composting materials were CFR and furfural slag. Therefore, the concentration in the initial composting sample was lower than that in the samples subjected to SE and thermal treatments. About 1200 mg/kg CEPC was detected in the initial composting samples. The residual antibiotic level was 50.2 mg/kg, representing a removal rate of 95.8%. Composting can therefore be used to degrade antibiotics via microorganisms. Bu [44] found that the gentamicin removal rate was over 98%, but the removal rate for all ARGs was only 53.2%. About 150.5 mg/kg CEPC remained after thermal treatment, representing a removal rate of only 90%. Similar results were also obtained by Chu [45], who found that with thermal treatment at 60 °C and 90 °C for 4 h, the antibiotic removal rate was persistently less than 90%. Meanwhile, CEPC was effectively degraded via SE treatment, with it no longer being detected after SE treatment. Temperature might be one of the most important factors in this process, with lower temperatures in the thermal treatment potentially not effectively damaging the molecular structure of cephalosporin. The high saturated vapor pressure involved in SE treatment corresponded to a high temperature of over 200 °C, which could break some of the lower-energy bonds in molecules such as the β-lactam ring [46]. In addition, through SE treatment, cells were disrupted and intracellular nutrients such as polysaccharides and proteins were released, which was more beneficial for the utilization of soil microorganisms [47].

The quantities of CEPC resistance genes and MGEs (*intI1* and *ISCR1*) all decreased remarkably during the SE and thermal processes (Figure 2b). Compared with the CFR, the total abundance of ARGs was reduced by 62.2% and 47.2% after the SE and thermal treatments, indicating that both of these treatments can effectively reduce the abundance of ARGs. It is worth noting that all of the ARGs exhibited similar decrease patterns. The levels of *intI1*, *ISCR1*, *ampC* and *blaCMY* all reduced by 33.3–90.0% and 13.3–66.7% after SE and thermal treatment, respectively, indicating that these physical approaches are not very selective for the removal of ARGs. As one of the most important efflux pumps genes [48], *tolC* was detected in the CFR, but its abundance was reduced by 60.0–88.0% after these three treatments. Chu et al. [45] found that only *tolC* was detected in CFR, and its abundance was reduced by 74.2% by radiation. Unlike SE and thermal approaches, ARGs subjected to composting showed different change patterns. The reasons behind this phenomenon might lie in the evolution of microbes involved in composting [49]. After composting, the total abundance of ARGs increased 1.4 times, while the levels of *intI1*, *ISCR1*, *ampC*, *blaCMY* and *blaSHV* all increased by 1.1–15 times. *ampC*, *blaCMY* and *blaSHV* belong to an important class of β-lactam antibiotics which encode β-lactamase [50]. These β-lactamases could inactivate β-lactam antibiotics by breaking the β-lactam ring [51]. It is speculated that the degradation mode of CEPC in composting might be the destruction of the β-lactam ring. This was also illustrated by the subsequent analysis of CEPC byproducts. In addition, the levels of *tolC*, *mecA*, *blaVIM* and *blaCTX* all decreased by 5.0% to 12.1%, indicating that microbes containing these genes are not suitable for the composting environment. The levels of *intI1* and *ISCR1* increased 1.2 and 1.1 times in the composting sample, indicating the risk of ARG transfer into the environment [52]. Composting may therefore be effective at degrading antibiotics (Figure 2a), but it is ineffective at removing ARGs.

### 3.3. The Loss of Nitrogen After SE, Composting and Thermal Treatments

**Figure 3:**
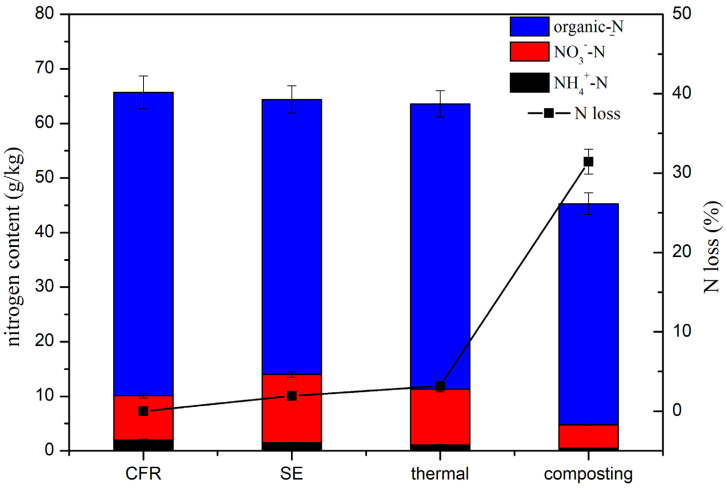
Changes in nitrogen distribution and nitrogen loss after SE, thermal, and composting treatments.

As shown in Figure 3, organic N was the main component of the TN in all of the samples. About 55.6 ± 0.5 g/kg organic N was observed in CFR, accounting for 84.6% of the TN. Mycelium is the main component of CFR. Therefore, the organic N mainly came from the mycelium. There was no significant change in NO_3_^−^ level after the SE and thermal treatments, while the NH_4_^+^-N level increased after the SE and thermal treatments. High temperatures could cause proteins to break down and produce NH_4_^+^-N [53]. A nitrogen loss of about 1.9% was obtained with the SE treatment, which was lower than that for thermal treatment, with a N loss of 3.2%. The possible reasons for this might be the longer duration of the thermal treatment, which could lead to the accumulation of more NH_4_^+^-N. Compared with the SE and thermal treatments, there was greater N loss after the composting procedure, reaching 31.4%. Composting is one of the most economic and efficient forms of manure management, but the emission of ammonia during composting is currently unavoidable [54]. The emission of ammonia was the major reason for N loss in this treatment, with ammonia accounting for 9.6–46% of the initial TN [55]. Ammonia emissions not only pollute the environment but also reduce the nutrient content in compost [56]. For high-quality fertilizers, the nitrogen content is an important indicator. During SE treatment, the shorter reaction time (only 15 min) and higher temperature help to significantly reduce the antibiotic levels in CFR, while retaining more nitrogen. Less N loss meant more nitrogen is preserved in the CFR, which is more beneficial for the growth of plants.

### 3.4. Antimicrobial Activity

**Figure 4:**
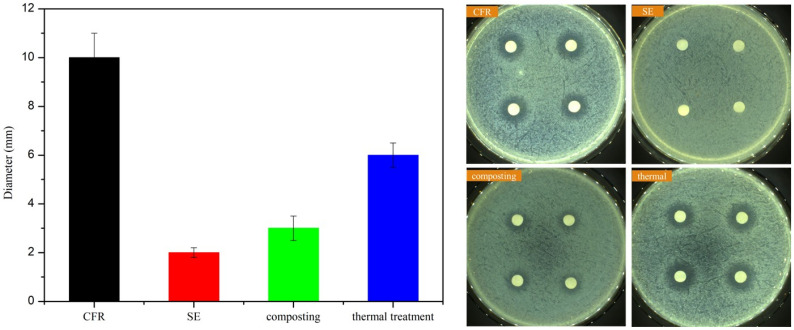
Changes in antimicrobial activity and inhibition circles against *S. aureus* after SE, composting, and thermal treatments.

β-lactam antibiotics mainly inhibit the cell walls of Gram-positive bacteria [57]. So, *S. aureus* was chosen for the measurement of antimicrobial activity. As seen in Figure 4, the antimicrobial activity was greatly reduced after each treatment. The inhibition zone was reduced by about 80.3%, 71.2% and 40.8% for the SE, composting, and thermal treatments. In particular, the minimum inhibitory diameter after SE was less than 2 mm, indicating that SE treatment was effective in eliminating antimicrobial activity. Over 90% of the antibiotics were removed in all three treatments (Figure 2a). However, the efficiency of antimicrobial activity loss was lower than that of CEPC removal. Similar results were also observed by Chu [45], who found that although antibiotic levels were reduced, the inhibition circle was reduced by only 40% via gamma radiation treatment. There may be two reasons for this phenomenon. On the one hand, the CEPC residue may still produce an inhibiting effect; on the other, the intermediate forms of CEPC might generate antimicrobial activity [58]. Therefore, it is important to further study these CEPC byproducts.

### 3.5. Identification of CEPC Intermediates and CEPC Degradation Pathways in the Three Treatments

**Figure 5:**
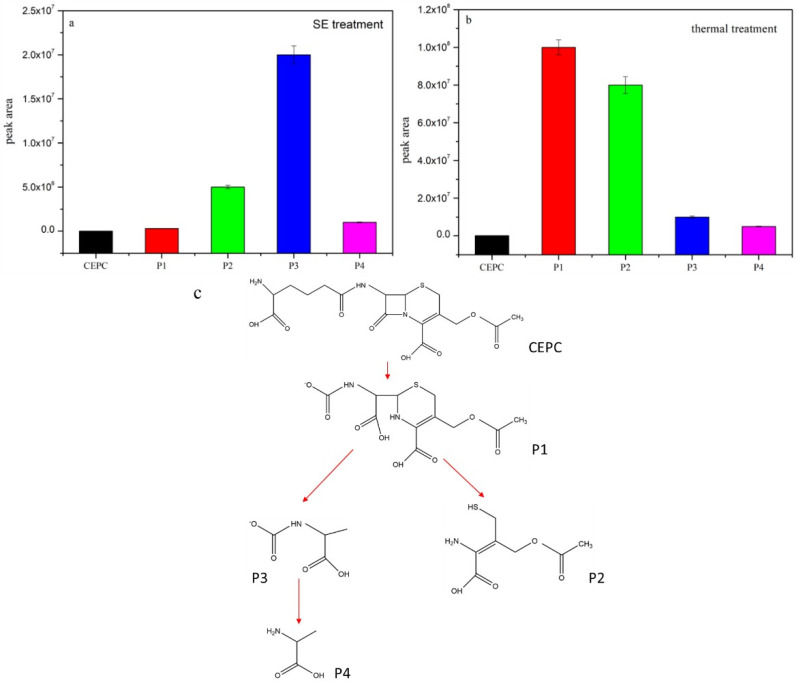
Peak areas of CEPC degradation products in SE treatment (**a**) and thermal treatment (**b**) and the potential degradation pathway in SE and thermal treatments (**c**).

The β-lactam ring and dihydrothiazine ring are considered to be the main pharmacophore in CEPC [21]. Four byproducts were identified via LC-MS analysis in both positive and negative mode after the SE and thermal treatments (Figure 5). The byproduct P1 with *m*/*z* 333 was identified as the β-lactam ring opening product, also being found after gamma irradiation and microwave treatments of CMD [21,59]. P2 with *m*/*z* 207 and P3 with *m*/*z* 133 were considered to be the dihydrothiazine ring cleavage products. P4 with *m*/*z* 89 was identified as alanine. Figure 5 reveals the peak area of different CEPC byproducts after SE and thermal treatments. In the SE treatment samples (Figure 5a), the main degradation products were P2 and P3, whose β-lactam ring and dihydrothiazine ring were totally destroyed. However, in the thermal treatment samples (Figure 5b), P1 became the major byproduct of CEPC, displaying damage only to the β-lactam ring. This indicates that the steam explosion treatment mainly destroyed both the β-lactam ring and the dihydrothiazine ring, while thermal treatment only damaged the β-lactam ring, while the dihydrothiazine ring was not degraded. Therefore, the inhibition zone was larger for the thermal treatment samples compared with that of the SE treatment samples.

**Figure 6:**
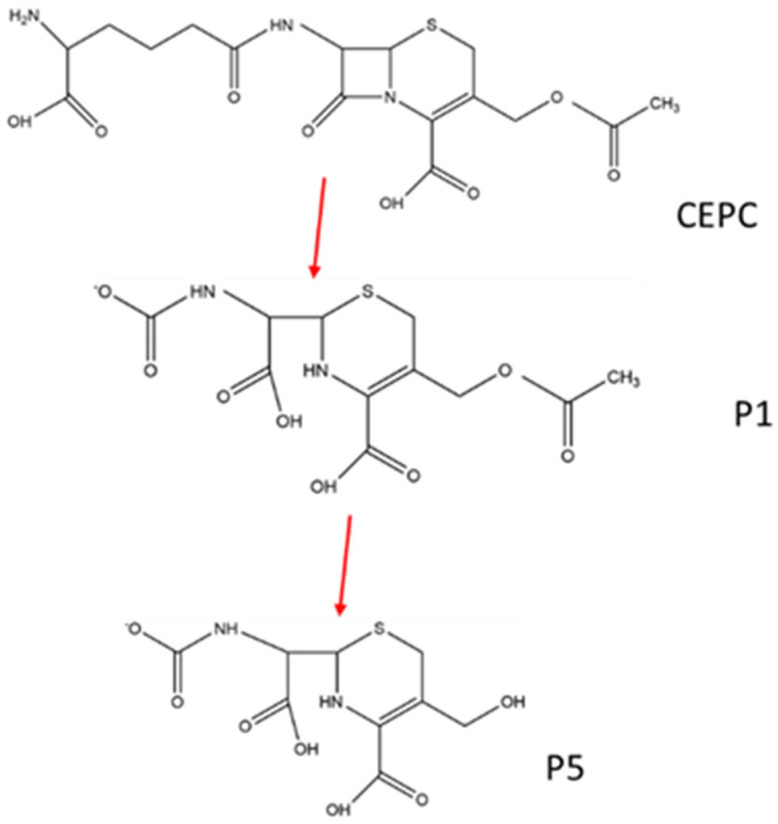
Predicted CEPC degradation pathway in composting.

A possible pathway of CEPC degradation in the SE and thermal treatments is proposed in Figure 5c based on the structure of CEPC byproducts. The major transformation processes of CEPC are β-lactam ring disruption, dihydrothiazine ring cleavage, and then amide bond breakage. Comparing these two physical treatments, the temperature might be the key factor. The temperature of the saturated vapor at a pressure of 1.5 MP in the SE treatment was over 200 °C, meaning it could break the binding bonds of the β-lactam and dihydrothiazine rings. Unlike physical treatment, the antibiotic degradation process in composting is mainly a biological process. P1 with *m*/*z* 333 and P5 with *m*/*z* 291 were observed in the composting treatment samples, whose dihydrothiazine rings were not broken. Meanwhile, the β-lactam ring can be degraded easily by β-lactamase in microorganisms [60]. A possible pathway for CEPC degradation in composting is shown in Figure 6. Composting produced CEPC byproducts with a dihydrothiazine ring which could still inhibit the growth of. *S. aureus*. These results indicate that compared with composting and thermal treatment, SE treatment has apparent advantages in the removal of both cephalosporin and its byproducts’ antimicrobial activity from CFR.

## 4. Discussion

Pruden [61] explicitly defined ARGs as a new type of environmental pollutant in 2006, which marked the official start of widespread academic attention being paid to ARG pollution. Since then, over the past 20 years, relevant research has developed rapidly, covering the sources, distribution, migration, and transformation of ARGs in soil, water, air, and other environmental media [62]. The persistent antibiotic residues in AFR act as a strong selective pressure on the surrounding environment, which can not only enrich ARGs in AFR itself, but also accelerate the horizontal transfer of ARGs among environmental microorganisms [63]. AFR is a typical solid waste generated during antibiotic production. It has dual properties: it is rich in proteins, carbohydrates, and other valuable nutritional components with high resource utilization potential, while it also bears high concentrations of antibiotic residues with great environmental risks. These contradictory characteristics have seriously restricted its resourceful application and safe disposal, making this a key and difficult point in the pollution control of the antibiotic manufacturing industry.

Among the three treatments evaluated in this study, SE exhibited significantly better overall performance than thermal treatment and composting. SE had a higher removal efficiency of antibiotic residues in CFR and could better retain proteins and other nutrient components with high resource value. In contrast, thermal treatment caused a serious loss of nutritional components from the sludge during the treatment process, while composting had the disadvantages of a long treatment cycle and high nutrient loss. Therefore, SE treatment seems to be a more efficient and feasible technical method for the harmless disposal and resource utilization of CFR.

Despite these promising results, several key questions remain unresolved and require further investigation to fully validate the feasibility of SE. Although SE effectively reduced antibiotic residue levels in CFR, trace amounts of ARGs, residual antibiotics, and their degradation products would still enter the soil environment once applied, which might induce the enrichment and horizontal transfer of ARGs in soil microorganisms and elevate the risk of ARG spread in the soil–plant system. Meanwhile, these CEPC byproducts (e.g., P2, P3, and P4) may still exhibit subtle toxicity to soil microorganisms, particularly beneficial bacteria (e.g., rhizobia) or fungi (e.g., mycorrhizae) that play critical roles in nutrient cycling. Future studies should evaluate the impact of SE-treated CFR on soil microbial community structure and function using high-throughput sequencing and functional assays. In addition, the increase in ammonium nitrogen (NH_4_^+^-N) levels after SE treatment may produce an inhibitory effect on seed germination or early seedling growth, especially for sensitive crops. Pot experiments and field trials are needed to determine the optimal application rate of SE-treated CFR, as well as its long-term effects on crop yield, quality, and nutrient uptake.

## 5. Conclusions

This study evaluated the feasibility of recycling CFR via three common treatments. Compared with composting and thermal treatments, SE treatment had significant advantages in the removal of CEPC and ARGs while reducing nitrogen loss. Meanwhile, the β-lactam ring and the dihydrothiazine ring in CEPC were mostly destroyed via SE treatment. SE treatment seems to be a promising option for addressing the challenges of removing antibiotics, antibiotic activity, and ARGs while preserving nitrogen. Therefore, SE treatment may have greater potential for the recycling of CFR.

## Figures and Tables

**Table 1:** Characteristics of cephalosporin fermentation dregs.

Parameters	CFR
pH	3.1 ± 0.1
Moisture (%)	70.5 ± 2.0
TC (g/kg)	460.5 ± 5.1
TN (g/kg)	65.4 ± 1.5
P (g/kg)	20.5 ± 0.5
K (g/kg)	5.6 ± 0.2

**Table 2:** Summary of LC-MS/MS analysis regarding cephalosporin and its degradation products during SE, thermal treatment, and composting treatment.

Compound	Retention Time (min)	Formula	Parent (*m*/*z*)	Daughters (*m*/*z*)
CEPC	5.06	C_16_H_21_N_3_O_8_S	415	356, 312, 185
P1	4.09	C_12_H_13_N_2_O_8_S	334	289, 221, 167
P2	2.20	C_7_H_13_NO_4_S	207	148, 59
P3	1.50	C_4_H_7_NO_4_	133	88, 45
P4	0.85	C_3_H_7_NO_2_	89	73
P5	2.85	C_8_H_10_N_2_O_7_S	292	261, 217, 175

**Table 3:** Primer sequences and expected amplicon size for each target gene considered.

Gene Name	Primer	Resistance Mechanism	Amplicon Size	Source
16S rRNA	F: CCTACGGGAGGCAGCAG R: ATTACCGCGGCTGCTGG	16S rRNA	194	[34]
*intI1*	F: TACCCGAGAGCTTGGCACCCA R: CGAACGAGTGGCGGAGGGTG	Integrase	312	[35]
*tolC*	F: GGCCGAGAACCTGATGCA R: AGACTTACGCAATTCCGGGTTA	efflux	64	[23]
*blaTEM*	F: AGCATCTTACGGATGGCATGA R: TCCTCCGATCGTTGTCAGAAGT	deactivate	101	[36]
*ampC*	F: TACCGCCTCTTGCTCCACAT R: TTTGCTGACCGAACCTAACT	deactivate	217	[35]
*blaSHV*	F: CTTTCCCATGATGAGCACCTTT R: TCCTGCTGGCGATAGTGGAT	deactivate	108	[37]
*blaCTX-M*	F: TTGGGTGATGAGACCTTCCG R: ACTGTGCCCGCTGAGTTTCC	deactivate	157	[37]
*blaSFO*	F: GCGGATGGAAATCAAACAAT R: TCACGCTTATCGCTGGGAAT	deactivate	258	[37]
*ISCR1*	F: CTTGCCAGGGCGTGAGGATA R: CGATTTGTCGGGCTTCTTGC	Integrase	382	[23]
*mecA*	F: GGTTACGGACAAGGTGAAATACTGAT R: TGTCTTTTAATAAGTGAGGTGCGTTAATA	protection	106	[37]
*cphA*	F: GCGAGCTGCACAAGCTGAT R: CGGCCCAGTCGCTCTTC	deactivate	168	[37]
*acrA*	F: TGGCGATGCCACCGTACT R: CAACGATCGGACGGGTTTC	efflux	62	[37]
*blaCMY*	F: AACTTGACGCCGAAGCCTAT R: TCAGCATCTCCCAGCCTAAT	deactivate	180	[37]
*blaVIM*	F: GCACTTCTCGCGGAGATTG R: CGACGGTGATGCGTACGTT	deactivate	135	[37]

## Data Availability

The datasets used and/or analyzed during the current study are available from the corresponding author on reasonable request.
